# The Inhibitory Effects of Djulis (*Chenopodium formosanum*) and Its Bioactive Compounds on Adipogenesis in 3T3-L1 Adipocytes

**DOI:** 10.3390/molecules23071780

**Published:** 2018-07-19

**Authors:** Charng-Cherng Chyau, Chin-Chen Chu, Shih-Ying Chen, Pin-Der Duh

**Affiliations:** 1 Research Institute of Biotechnology, Hungkuang University, 34 Chung-Chie Road, Shalu County, Taichung 43302, Taiwan; ccchyau@hk.edu.tw; 2 Department of Anesthesiology, Chi-Mei Medical Center, Tainan 71004, Taiwan; chinchen.chu@gmail.com; 3 Department of Health and Nutrition, Chia Nan University of Pharmacy and Science, Tainan 71710, Taiwan; shihying@mail.cnu.edu.tw; 4 Department of Food Science and Technology, Chia Nan University of Pharmacy and Science, 60 Erh-Jen Road, Section 1, Pao-An, Jen-Te District, Tainan 71710, Taiwan

**Keywords:** Djulis (*Chenopodium formosanum*), adipogenesis, lipid accumulation, rutin, kaempferol, betanin

## Abstract

The aim of this study was to provide new insights into the role of the ethanolic extracts of Djulis (*Chenopodium formosanum*, EECF) and its bioactive compounds in preventing adipogenesis in 3T3-L1 adipocytes. The results demonstrated EECF significantly inhibited oil red O-stained material (OROSM), triglyceride levels and glycerol-3-phosphate dehydrogenase (GPDH) activity in 3T3-L1 adipocytes. The expression of the critical molecules involved in lipid synthesis such as PPARγ, C/EBPα and SREBP-1c was attenuated in EECF-treated cells. According to HPLC-DAD and HPLC-MS/MS analysis, rutin, kaempferol, betanin and another nine compounds were present in EECF. The suppression of lipid accumulation by rutin, kaempferol and betanin occurred by decreasing the gene expression of PPARγ, C/EBPα and SREBP-1c. Taken together, these findings suggest the presence of bioactive compounds in EECF may partly account for the anti-adipogenesis of EECF and EECF is therefore a potentially lipid lowering functional food.

## 1. Introduction

Overweightness and obesity are defined by the World Health Organization (WHO) as an abnormal or excessive fat accumulation that may impair health. The WHO survey indicates, worldwide obesity has nearly tripled since 1975. Further, in 2016, more than 1.9 billion adults, 18 years and older, were overweight. Of these over 650 million were obese [1]. The WHO survey indicates, in 2008, more than 1.4 billion adults were overweight and were than half a billion were obese [1]. Overweightness and obesity are caused by the increase in the size and the amount of fat cells in the body. It has been reported obese humans are at increased risk of developing metabolic syndrome, hypertension, heart disease, diabetes, high blood cholesterol, cancers and sleep disorders [2]. Therefore, preventing, controlling and treating excess body weight and obesity are becoming more urgent. Many different effective approaches have been suggested for treating and controlling obesity, including diet therapy, exercise, and pharmacological agents [2,3]. The pharmacological agents can reduce or control weight by altering appetite, metabolisms or the consumption of calories [3]. However, the undesirable side effects of the anti-obesity drugs outweigh their beneficial effects [3]. Therefore, the search for complementary and alternative therapies with functional biomaterials capable of regulating the lipid metabolism and preventing obesity is of great concern for scientists and researchers. Epidemiological studies have shown increased intakes of the dietary phytochemicals present in natural sources are associated with a lower risk of human diseases [4]. Consequently, there has been a substantial increase in studies of the biological effects of natural plants and their derived compounds in preventing and treating overweightness and obesity.

Djulis (*Chenopodium formosanum*) is traditionally used as a native cereal and as one of the ingredients of local wine brewed by aboriginal people in Taiwan. Many studies have reported Djulis contains high levels of starch, dietary fiber, proteins, and grain-limited essential amino acids (e.g., lysine) [5]. Therefore, Djulis has received more and more attention in the use of functional foods. With respect to the biofunctional effects of Djulis, previous studies by the authors reported Djulis showed significant hepatoprotective activity in vivo and in vitro due to ameliorating oxidative damage [6,7]. The antioxidant potential of Djulis has also been reported [8]. Apart from these, Djulis can protect the skin against UV-induced damage [5]. Apparently, the biological effects of Djulis, mentioned above, are attributed to the bioactive compounds present in Djulis [6,7]. Given Djulis demonstrated biological effects [6,7,8] such as antioxidant, hepatoprotection, and skin protection, it is also possible fat accumulation may be regulated by Djulis. However, no reports have been found so far on the effectiveness of Djulis in regulating fat accumulation. Further, in vitro and in vivo studies by the authors show the bioactive compounds present in Djulis have phytochemical characteristics and biological effects, or a combination of the two. Therefore, this study aims to determine whether Djulis and its bioactive compounds can inhibit adipogenesis in 3T3-L1 adipocytes, in vitro.

## 2. Results

### 2.1. Effects of EECF on 3T3-L1 Viability

To understand whether EECF affected the 3T3-L1 growth, 3T3-L1 cells were allowed to differentiate into adipocytes for 8 days, and then we examined the cytotoxicity of EECF toward 3T3-L1 adipocytes. The mature cells were incubated for 72 h in medium with various concentrations of EECF (10–500 μg/mL), and the cell growth was determined using MTT assay. The cell viability in the presence of EECF at 10, 100, 250, and 500 μg/mL was 95.4%, 98.9%, 105.8%, and 99.1%, respectively, indicating EECF did not affect cell viability over a wide range of concentration. However, in medium containing 250 μg/mL EECF, cell viability slightly increased to approximately 5.78%. These results indicated EECF does not affect cell viability and is not cytotoxic to 3T3-L1 adipocytes.

### 2.2. EECF Attenuates Lipid Accumulation in 3T3-L1 Adipocytes

**Figure 1:**
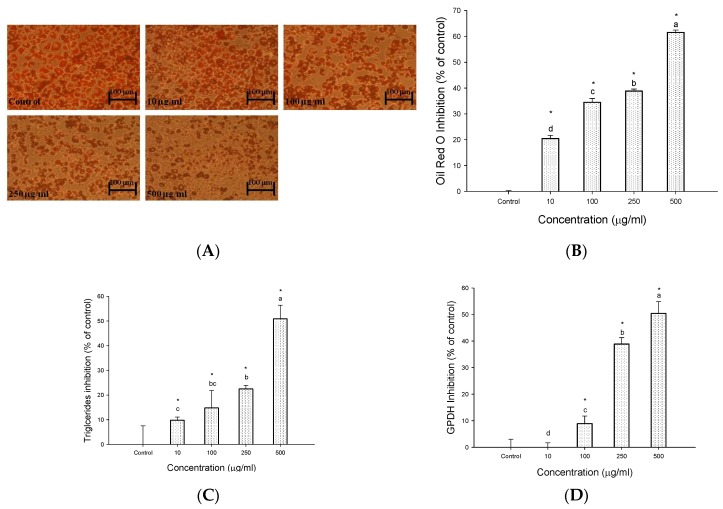
Effect of ethanol extracts of Djulis (EECF) on adipogenesis. (**A**) The microscopic images of Oil red O-staining of EECF-treated 3T3-L1 cells. (**B**) Oil red O-staining of EECF-treated 3T3-L1 cells. 3T3-L1 cells were incubated with 10–500 μg/mL of EECF for 72 h at 37 °C in 5% CO_2_ incubator. (**C**) EECF-mediated suppression of intracellular triglyceride levels of 3T3-L1 cells. 3T3-L1 cells were incubated with 10–500 μg/mL of EECF for 72 h at 37 °C in 5% CO_2_ incubator. (**D**) Effect of EECF on GPDH activity in 3T3-L1 cells. The cells were incubated with 10–500 μg/mL of EECF for 72 h at 37 °C in 5% CO_2_ incubator. Data are expressed as the mean ± SD of three independent experiments. * (*p* < 0.05), compared to the control. Values in each concentration with different superscript letters are significantly different (*p* < 0.05).

The effects of EECF on lipid accumulation in 3T3-L1 adipocytes are shown in Figure 1. The microscopic images of oil red O-stained adipocytes are shown in Figure 1A. The number of lipid droplets in the differentiated cells when EECF was present was decreased compared with the control. Quantitatively, OROSM showed 10, 100, 250 and 500 μg/mL EECF reduced lipid accumulation by 20.4, 34.5, 38.9 and 61.6%, respectively (Figure 1B). In addition, further analysis of the effects of EECF on the triglyceride levels are shown in Figure 1C. It was observed that 10, 100, 250 and 500 μg/mL EECF reduced the triglyceride levels in 3T3-L1 adipocytes by 9.8, 14.8, 22.5 and 50.9%, respectively, indicating in the presence of 10–500 μg/mL EECF, the intracellular triglyceride level was dramatically decreased in a concentration-dependent manner. Meanwhile, the effect of EECF on GPDH activity is shown in Figure 1D. The intracellular GPDH activity was concentration-dependently lowered in cells treated with EECF. The GPDH activity of the cells treated with EECF at 500 μg/mL was only about half that of the control. These observations clearly indicate EECF suppressed the accumulation of intracellular lipids in 3T3-L1 adipocytes.

### 2.3. EECF Inhibited Lipid Accumulation by Regulating Adipogenic Genes

**Figure 2:**
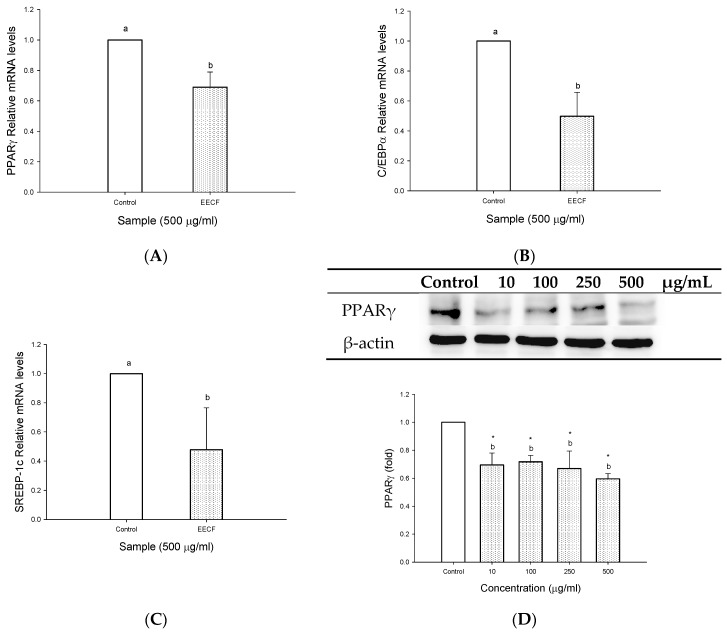
Effect of ethanol extracts of Djulis (EECF) on expression of adipogenic genes in 3T3-L1 adipoctyes. (**A**) The effect of EECF on gene levels of PPARγ in 3T3-L1 cells. The cells were incubated with EECF for 6 h. (**B**) The effect of EECF on gene levels of C/EBPα in 3T3-L1 cells. The cells were incubated with EECF for 12 h. (**C**) The effect of EECF on gene levels of SREBP-1c in 3T3-L1 cells. The cells were incubated with EECF for 12 h. (**D**) The effect of EECF on PPARγ protein expression in 3T3-L1 cells. The cells were incubated with EECF for 18 h. Data are expressed as the mean ± SD of three independent experiments. * (*p* < 0.05), compared to the control. Values in each concentration with different superscript letters are significantly different (*p* < 0.05).

Transcription factors such as PPARγ, C/EBPα and SREBP-1c are involved in adipocyte differentiation. To understand the molecular mechanism for inhibiting lipid accumulation in adipocytes caused by EECF, the effect of EECF on the gene expression involved in lipid biosynthesis and degradation was conducted. Of the concentrations used, EECF at 500 μg/mL demonstrated the highest decrease in OROSM, triglyceride levels, and GPDH activity in the cells. Therefore, 500 μg/mL was used to determine gene expression. As shown in Figure 2, the effect of EECF at 500 μg/mL on the expression of PPARγ (Figure 2A), C/EBPα (Figure 2B) and SREBP-1c (Figure 2C) by quantitative PCR analysis. As expected, in the EECF-treated adipocytes, PPARγ, C/EBPα and SREBP-1c mRNA levels were significantly inhibited. In addition, a decrease in protein levels of PPARγ was also observed due to the direct relationship between mRNA and protein levels (Figure 2D). Thus, EECF suppressed the expression of adipogenic genes in the adipocytes. This observation indicates EECF was able to regulate the expression of adipogenic genes at early stage of adipogenesis, thereby inhibiting lipid accumulation in 3T3-L1 cells.

### 2.4. Analysis of Bioactive Compounds Present in EECF

**Figure 3:**
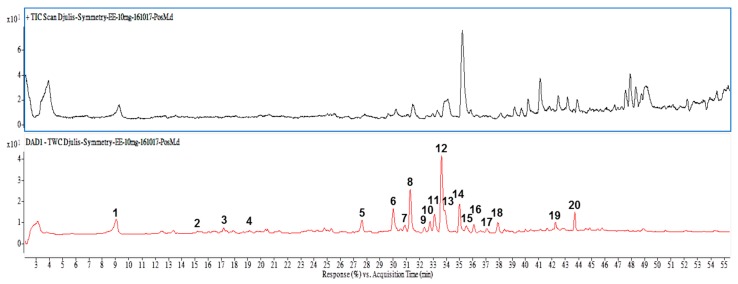
Total ion chromatograms of LC/MS analysis in positive electrospray ionization (**top panel**) and HPLC-photodiode array detection chromatograms (**lower panel**) at full scan of 210–600 nm from aqueous ethanol extract of Djulis. Peak numbers are referred to Table 1.

In our previous work, bioactive compounds such as rutin, kaempferol, betanin and another nine compounds were identified in the water extracts of Djulis [6]. However, when extracting natural compounds from plants, the contents of bioactive compounds may vary due to differences in the climate, harvest and storage conditions as well as their extractability by solvents. In the current work, Djulis was extracted with ethanol. It was therefore necessary to analyze and identify the bioactive compounds of ethanol extracts of Djulis in the present work. Figure 3 shows the HPLC-MS total ion and HPLC-DAD chromatogram for EECF. Twelve compounds were identified by comparison with authentic samples (Table 1). Of these compounds, nine were phenolic compounds, including protocatechuic acid derivatives (**1**), quercetin derivatives (**5**), quercetin-3-*O*-rutinoside-7-*O*-rhamnoside (**6**), quercetin-3-*O*-deoxy-hexose-*O*-hexose-*O*-pentose (**7**), camellianoside (**8**), kaempferol derivative (**9**), kaempferol-3-*O*-[6′″-*p*-coumaroyl-glucosyl-β-(1→4)-rhamnoside] (**10**), quercetin-3-*O*-2″-(6″-*p*-coumaroyl)-glucosylrhamnoside (**11**), rutin (**12**). Apart from these, amaranthine (**2**), betanin (**3**) and isobetanin (**4**) which are responsible for the red colors in Djulis are present in EECF.

Biological capacity is associated with the levels and composition of the bioactive compounds present in food. According to the data obtained, the identified compounds present in EECF mostly consist of flavonoid glycosides and free flavonoids, such as kaempferol derivatives and quercetin derivatives and rutin. Among the compounds identified, rutin is the most abundant compound in EECF. In the previous work by the authors, the protective effect of the water extracts of Djulis against oxidative damage in vitro and in vivo is partly associated with betanin, rutin, kaempferol and other uncharacterized compounds present in the water extracts of Djulis. Therefore, in the current work, rutin, betanin and kaempferol are selected as reference compounds for further exploring the role of bioactive compounds in EECF in suppressing lipid accumulation in 3T3-L1 adipocytes.

**Figure 4:**
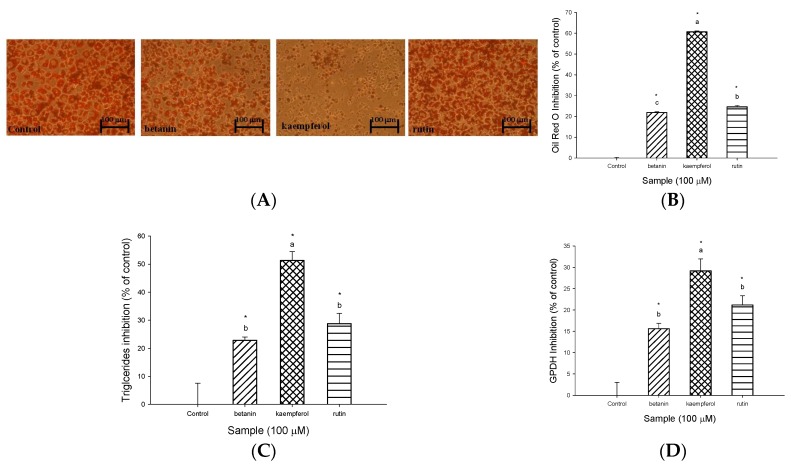
Effects of rutin, kaempferol and betanin on adipogenesis in 3T3-L1 cells. (**A**) The microscopic images of Oil red O-staining of rutin-, kaempferol- or betanin-treated 3T3-L1 cells. (**B**) Oil red O-staining of rutin-, kaempferol- or betanin-treated 3T3-L1 cells. 3T3-L1 cells were incubated with 100 μM of rutin, kaempferol or betanin, respectively, for 72 h at 37 °C in 5% CO_2_ incubator. (**C**) Rutin-, kaempferol- or betanin-mediated suppression of intracellular triglyceride levels of 3T3-L1 cells. 3T3-L1 cells were incubated with 100 μM of rutin, kaempferol or betanin, respectively, for 72 h at 37 °C in 5% CO_2_ incubator. (**D**) Effect of 100 μM of rutin, kaempferol or betanin on GPDH activity in 3T3-L1 cells. The cells were incubated with 10–500 μg/mL of rutin, kaempferol or betanin, respectively, for 72 h at 37 °C in 5% CO_2_ incubator. Data are expressed as the mean ± SD of three independent experiments. * (*p* < 0.05), compared to the control. Values in each concentration with different superscript letters are significantly different (*p* < 0.05).

To understand whether bioactive compounds such as rutin, kaempferol and betanin show any cytotoxicity towards 3T3-L1 adipocytes, the cytotoxicity of rutin, kaempferol and betanin in 3T3-L1 adipocytes is measured using an MTT assay. None of the bioactive compounds, rutin, kaempferol and betanin, in the range from 10–100 μM show any cytotoxicity effect on the survival of 3T3-L1 adipocytes (data not shown). We next explored which bioactive compounds account for the suppression of lipid accumulation in 3T3-L1 adipocytes, and the effects of rutin, kaempferol and betanin on OROSM, triglyceride levels and gene expression in 3T3-L1 adipocytes were determined. Figure 4 shows the effects of rutin, kaempferol and betanin on lipid accumulation in 3T3-L1 adipocytes. As expected, the microscopic images of oil red O-stained adipocytes treated with rutin, kaempferol and betanin indicating the number of lipid droplets in the differentiated cells had decreased (Figure 4A). In addition, the treatment of cells with rutin, kaempferol and betanin in the range of 10–100 μM significantly inhibited OROSM (Figure 4B). In response to 100 μM rutin, kaempferol and betanin-treatment, 24.6, 60.7 and 21.9% inhibition of lipid accumulation were observed. In addition, treating with rutin, kaempferol and betanin at 100 μM resulted in 28.8, 51.3 and 22.9% inhibition of the triglyceride levels, respectively (Figure 4C). Figure 4D shows the effect of 100 μM of rutin, kaempferol and betanin on GPDH activity in 3T3-L1 adipocytes. Apparently, rutin, kaempferol and betanin at 100 μM also significantly reduced the GPDH activity in 3T3-L1 adipocytes.

**Figure 5:**
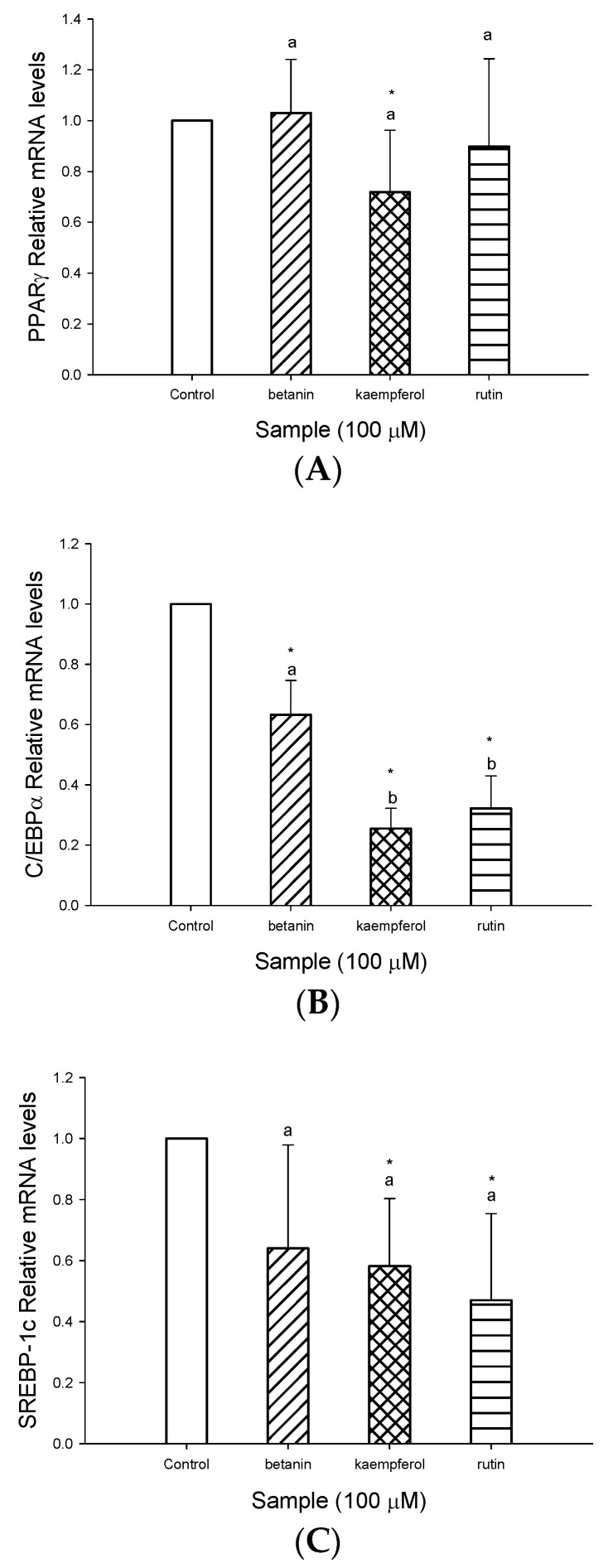
Effects of of rutin, kaempferol and betanin on expression of adipogenic genes. (**A**) The effect of rutin, kaempferol and betanin on gene levels of PPARγ in 3T3-L1 cells. The cells were incubated with rutin, kaempferol or betanin, respectively, for 6 h. (**B**) The effect of rutin, kaempferol and betanin on gene levels of C/EBPα in 3T3-L1 cells. The cells were incubated with rutin, kaempferol or betanin, respectively, for 12 h. (**C**) The effect of rutin, kaempferol and betanin on gene levels of SREBP-1c in 3T3-L1 cells. The cells were incubated with rutin, kaempferol or betanin, respectively, for 12 h. Data are expressed as the mean ± SD of three independent experiments. * (*p* < 0.05), compared to the control. Values in each concentration with different superscript letters are significantly different (*p* < 0.05).

The effect of rutin, kaempferol and betanin on the expression of adipogenic genes were determined (Figure 5). Real-time PCR data shows kampferol at 100 μM significantly suppressed PPARγ mRNA expression (Figure 5A). In addition, exposure of adipocytes to rutin, kaempferol and betanin at 100 μM significantly down-regulated C/EBPα mRNA expression (Figure 5B). For SREBP-1c, kaempferol and rutin at 100 μM significantly reduced SREBP-1c mRNA expression in comparison to adipocytes differentiating without bioactive compounds treatment. Betanin at 100 μM suppressed SREBP-1c mRNA expression, but no significant difference was found between betanin and the control (Figure 5C). These findings indicate the three bioactive compounds were significantly down-regulating C/EBPα and SREBP-1c mRNA expression. Therefore, rutin, kaempferol and betanin may be mediators of lipolysis and adipocyte accumulation, leading to inhibiting lipogenesis in 3T3-L1 adipocytes.

## 3. Discussion

Treating obesity with drugs often has undesirable side effects and weight gain rebounds when the application of medications is discontinued [17]. Therefore, more research on health foods is required to develop novel treatments for the prevention and effective therapy of obesity. It has been reported that many plant-derived nutraceutical/functional food ingredients demonstrate significant alleviation of obesity and associated complications [3]. According to the previous studies by the authors, the water extracts of Djulis provided significant protection against cytotoxicity induced by oxidative stress in vitro [7] and in vivo models [6]. In this regard, we speculated Djulis could demonstrate other remarkable biological effects, in addition to the protective effect against hepatotoxicity. Therefore, this study further explored whether EECF and its bioactive compounds would demonstrate an in vitro antiadipogenic effect in 3T3-L1 adipocytes. According to the data obtained, EECF suppressed the accumulation of intracellular lipid droplets and repressed the expression of adipogenic genes in 3T3-L1 adipocytes. The amount of lipid accumulation and inhibition of adipogenesis can be decreased by deletion of adipocytes via apoptosis as well as by inhibiting adipogenesis of the cells [18,19]. The results obtained indicates EECF in the range from 10–500 μg/mL showed no cytotoxic effect on 3T3-L1 adipocytes growth, at least up to 500 μg/mL the highest concentration tested, thus indicating EECF had no apoptotic activity toward adipocytes [18]. 3T3-L1 cells induced by a chemical cocktail differentiate to form adipocytes, with the accumulation of triglycerides as one of the hallmarks of adipogenesis. Thus, the suppression of triglyceride formation in 3T3-L1 adipocytes is regarded as an anti-obesity effect [20]. As shown in Figure 1C, the 3T3-L1 adipocytes exposed to EECF showed a decreased triglycerides level in cells. In addition, cell number analysis of 3T3-L1 adipocytes treated with EECF was conducted by OROSM. The results show EECF significantly decreases the OROSM in 3T3-L1 adipocytes. GPDH is an important biochemical marker for the differentiation of preadipocyte to adipocyte. It was found GPDH activity had a very significant positive correlation with the main synthesized fatty acids in the cells [21]. In this study, treatment with EECF led to decreased GPDH activity (Figure 1D). These results clearly imply EECF significantly decreased fat accumulation in 3T3-L1 adipocytes.

The PPARγ and C/EBPα have emerged as master regulations of adipogenesis [22], therefore, to understand whether EECF inhibited lipid accumulation through regulating PPARγ, C/EBPα, real-time RT-PCR was conducted to determine the levels of the adipocyte differentiation-related gene in 3T3-L1 adipocytes. Our data revealed EECF suppressed gene expression of PPARγ and C/EBPα. PPARγ and C/EBPα play important roles in the early stage of adipocytes differentiation and are transcriptional factors for many genes [23]. PPARγ is a potent inducer of adipogenesis, in fact, it can promote the transdifferentiation of culture myoblasts to adipocytes, particularly when coexpressed with C/EBPα [23,24]. In addition, C/EBPα is a key transcriptional factor in adipogenesis and its expression is activated by C/EBPβ, C/EBPδ and PPARγ in adipocytes [18]. According to the results obtained, the cells treated with EECF result in suppression of gene expression of PPARγ and C/EBPα, indicating the decreased expression of both genes contributed to suppressed differentiation of the preadipocytes into adipocytes [19]. In other words, EECF inhibited adipogenesis by regulating the transcriptional factor cascade upstream of PPARγ and C/EBPα expression [24]. In addition, SREBP-1c is a key lipogenic transcriptional factor, which directly activates the expression of more than 30 genes, such as FAS, ATP-citrate lyase (ACL), acetyl-CoA carboxylase (ACC), stearoyl-CoA desaturase (SCD), and glyceraldehydes-3-phosphate acyltransferase (GPAT), dedicated to fatty acid biosynthesis and uptake of fatty acids, cholesterol and triglycerides [2,25,26]. However, overexpression of SREBP-1c in 3T3-L1 cells in the presence of hormonal inducement of differentiation results in elevated adipocyte marker expression and lipid accumulation [23]. 5′-Adenosine monophosphate-activated protein kinase (AMPK) is a multisubunit enzyme recognized as a major regulator of lipid biosynthetic pathways, serving as a metabolic master switch response to alterations in cellular energy change [26]. Although the influence of EECF and the three bioactive compounds on the up-regulation of AMPK activation was not conducted in this study, many reports noted that rutin decreased high fat diet-induced body weight gain through peroxisome proliferator-activated receptor gamma coactivator 1-alpha (PGC-1α) and AMPK activation accompany with the decrease of adipogenesis in adipose tissue [27]. It was found rutin is a bioactive compound present in EECF. Therefore, we suggest EECF could stimulate AMPK activity, therefore decreasing adipogenesis in 3T3-L1 cells. However, further experiments are necessary to validate its effect on regulating AMPK activity. In addition, previous studies showed polyphenolic extracts from many plants can activate AMPK, prevent SREBP-1c translocation to the nuclei and finally inhibit FAS expression [26]. Current data shows the expression of SREBP-1c was reduced in response to EECF, indicating EECF suppressed gene expression of SREBP-1c in 3T3-L1 adipocytes. It is speculated EECF may activate AMPK and then prevent SREBP-1c translocation to the nuclei, thereby leading to inhibition of FAS expression and finally inhibition of lipogenesis.

Many studies have reported that phytochemical and biological compounds identified from natural sources play important roles in protecting against oxidative stress, inflammation, carcinogenesis and other human diseases. These compounds have recently received much interest in the search for natural bioactive compounds that contribute to anti-obesity properties. For example, diallyl trisulphide, a garlic-derived organo-sulphur compound, inhibited adipogenesis in 3T3-L1 adipocytes through regulating the gene levels of lipogenesis, lipolysis and adipokines [28]. Phillyrin prevents lipid accumulation in HepG2 cells by blocking the expression of SREBP-1c and FAS through LKB1/AMPK activation [29]. Caffeine had anti-obesity effects by suppressing body weight gain and adipose tissue formation [30]. Rutin could attenuate lipid accumulation by decreasing lipogenesis and oxidative stress in hepatocyte [26]. In the current study, rutin, kaempferol, betanin and another nine compounds were identified and present in EECF. To compare with previous report [6], rutin was found to be significantly existed in the ethanol extract in the study than that in water extract. It was worth noting that one of the bioactive compound of 20-hydroxyecdysone (peak No. 14) did not detected in the water but in the ethanol extract [6]. In an attempt to elucidate which bioactive compounds such as rutin, kaempferol and betanin identified in EECF induced the suppression of lipogenesis of 3T3-L1 adipocytes, the effects of the bioactive compounds on lipid accumulation and regulation of the gene levels of lipogenesis in 3T3-L1 adipocytes were examined. As expected, rutin, kaempferol and betanin inhibited OROSM, triglyceride levels and GPDH activity in 3T3-L1 adipocytes. Therefore we suggest EECF demonstrates marked inhibition of lipid accumulation towards 3T3-L1 adipocytes probably in part due to the rutin, kaempferol and betanin present in WECF. For expression of the gene level, adipose tissue is able to produce and secrete a PPARγ and C/EBPα, which are transcriptional factors playing roles in the early stage of adipocyte differentiation [31]. In addition, another transcription factor induced very early during adipocyte differentiation is SREBP-1c and differentiation factor 1 (ADD 1), which participate in adipocyte gene expression [32]. Interestingly, rutin, kaempferol and betanin treatment suppressed the expression of C/EBPα and SREBP-1c expression, and kaempferol treatment reduced the PPARγ expression, indicating rutin, kaempferol and betanin could inhibit adipogenesis by regulating the transcriptional factor cascade upstream of SREBP-1c and C/EBPα and PPARγ expression [31]. In addition, SREBP-1c/ADD1 can increase fatty acid and fat synthesis, and this has partly been attributed to its proposed influence on PPARγ activity [32]. Our data showed rutin, kaempferol and betanin were able to suppress the gene expression of SREBP-1c, implying rutin, kaempferol and betanin may prevent these transcriptional factors from translocating to the nuclei and down-regulating SREBP-1c expression, thereby inhibiting lipogenesis [2]. In addition, according to above results, we found EECF decreased lipid accumulation, in parallel with the inhibition of lipid accumulation and gene expression by rutin, kaempferol and betanin, indicating these bioactive compounds may account for inhibition of lipogenesis of EECF. With respect to the effect of bioactive compounds on lipid accumulation, quercetin-rich formulation supplemented rats demonstrated much less lipid accumulation and smaller sized adipocytes [33]. In addition, the intake of protocatechuic acid significantly diminished the activity and mRNA expression of SREBP-1c and stearoyl-CoA desaturaes-1 and decreased hepatic lipid accumulation [34]. Babootar et al. noted combined treatment with genestein, quercetin and resveratrol demonstrated remarkably higher inhibition of adipogenesis than individual molecules [3]. According to the analysis of HPLC-DAD and HPLC/ESI-MS/MS, there are eight compounds, along with rutin, kaempferol and betanin and other unidentified peaks in EECF, which may contribute to the inhibition of lipogenesis due to a direct action or the synergy between the compounds, thereby forming the inhibition of lipogenesis. In other words, the inhibitory effect of EECF on lipogenesis in 3T3-L1 adipocytes might be attributed to a direct effect and also the synergistic effect of a combination of bioactive compounds present in EECF [6].

Obesity, metabolic syndrome and type 2 diabetes mellitus are strictly related and are key pathogenetic factors in non-alcoholic fatty liver disease (NAFLD) [35]. A previous work by the authors demonstrated the hepatoprotective effect of Djulis is attributed to rutin, kaempferol, and betanin present in Djulis. According to the results obtained, EECF and its bioactive compounds, rutin, kaempferol, and betanin, demonstrated anti-adipogenesis in 3T3-L1 cells. Therefore, we speculated EECF and its bioactive compounds may be of benefit for preventing NAFLD. However, this speculation requires further study in vivo.

## 4. Materials and Methods

### 4.1. Materials

3T3-L1 cell (BCRC Number: 60159) was obtained from the Bioresource Collection and Research Center (BCRC, Food Industry Research and Development Institute, Hsinchu, Taiwan). Oil red O solution, 3-isobutyl-1-methylxanthine, dexamethasone, insulin, betanin, rutin and kaempferol were obtained from Sigma (St. Louis, MO, USA). The dye MTT (3-[4,5-dimethylthiazol-2-yl]-2,5-diphenyltetrazolium bromide) was purchased from PanReac AppliChem (Darmstadt, Germany). Dulbecco’s modified Eagle’s medium (DMEM), Trypsin-EDTA, penicillin-streptomycin, bovine calf serum, and fetal bovine serum were purchased from Gibco (Grand Island, NY, USA). Anti-PPARγ and anti-β-actin were purchased from Cell Signaling Technology (Danvers, MA, USA). All other chemicals were reagent grade.

### 4.2. Sample Preparation

Djulis (*Chenopodium formosanum*), identified by Professor Yau-Lun Kuo of the Department of Forestry, National Pingtung University of Science and Technology, was purchased from Kullku Farm, Pingtung, Taiwan, and was ground to a fine powder before the extraction. The voucher specimen (No. CNU-101) was deposited in the Department of Food Science and Technology, Chia Nan University of Pharmacy and Science. The powder (10 g) was extracted with 50% ethanol (100 mL) and stirred for 16 h, at room temperature. The extract was filtered and the residue was re-extracted under the same conditions. The combined filtrates were evaporated to dryness for ethanol under vacuum and weighed to determine the yield of soluble constituents. The ethanolic extract of Djulis, abbreviated as EECF, was stored at 4 °C until used.

### 4.3. Cell Culture

3T3-L1 preadipocytes were planted into 6-well plates and maintained in DMEM supplemented with 10% bovine calf serum at 37 °C in a humidified 5% CO_2_ incubator. Adipocytic differentiation was induced by the differentiation medium A (0.5 mM 3-isobuty1-1-methylxanthine, 1 µM dexamethasone, 1 µM insulin, 1.5 g/L sodium bicarbonate, 10% fetal bovine serum and 1% antibiotic dissolved in high glucose DMEM) that were added to culture medium in every 48 h for 4 day. Afterwards, the medium was changed to differentiation medium B (1 µM insulin, 1.5 g/L sodium bicarbonate, 10% fetal bovine serum and 1% antibiotic dissolved in high glucose DMEM) and was freshly replaced every day. The cells were harvested 8 days after the initiation of differentiation [31].

### 4.4. Cell Viability Assay

Cell viability was assessed by the MTT viability assay [31]. The culture media containing test samples (10–500 μg/mL) was added to each well and the cells were incubated for 72 h, with untreated cells served as control. Then, culture media was replaced with MTT (0.5 mg/mL) and incubated in the dark for 1 h. Formazan crystals were solubilized (100% DMSO), and absorbance was measured using a micro-plate reader (Thermo Multiskan Spectrum, Waltham, MA, USA) at 540 nm.

### 4.5. Oil Red O-Staining of 3T3-L1 Adipocytes

Intracellular lipid accumulation was measured using Oil red O [20]. Freshly 60 mL Oil red O solution (0.5% in isopropanol) and 40 mL deionized water was prepared for the Oil red O working solution. Cells were incubated with test samples for 72 h at 37 °C in a humidified 5% CO_2_ incubator. Cells were washed twice with phosphate buffered saline (PBS, pH 7.4) and then fixed with 10% neutral buffered formalin for at least 20 min at room temperature. After fixation, cells were washed twice with PBS and stained with the Oil red O working solution for 60 min. The image of stained cells were photographed, and then eluted stained Oil red O by adding 100% isopropanol, finally, O.D. was measured at 510 nm in spectrophotometer (Thermo Multiskan Spectrum).

### 4.6. Quantification of Intracellular Triglyceride

Cells were incubated with test samples for 72 h at 37 °C in a humidified 5% CO_2_ incubator. Cells were collected and lysed in lysis buffer (1% Triton X-100 in PBS) for 30 min on ice box. The total triglyceride content in cells was determined using a commercial triglyceride assay kit (Cayman Chemical, Ann Arbor, MI, USA). The protein concentration was estimated with the Pierce^TM^ BCA protein assay kit (Thermo Scientific, Rockford, IL, USA) using bovine serum albumin as a standard. Inhibition (%) was expressed as percent decrease in triglyceride content against control (0%).

### 4.7. Determination of Glycerol-3-Phosphate Dehydrogenase Activity

3T3-L1 adipocytes were harvested 8 days after the initiation of differentiation. Cells were incubated with 10–500 μM of test samples for 72 h at 37 °C in a humidified 5% CO_2_ incubator. Cells were washed twice carefully with ice-cold PBS on 3T3-L1 adipocytes, and lysed in 25 mM Tris/1 mM EDTA, pH 7.5 for the measurement of glycerol-3-phosphate dehydrogenase (GPDH) specific activity. GPDH activity was determined by Glycerol-3-Phosphate Dehydrogenase Activity Colorimetric Assay Kit, (Biovision, Milpitas, CA, USA). Protein concentration was determined by the Pierce^TM^ BCA protein assay kit (Thermo Scientific) using bovine serum albumin as a standard. Enzyme activity was expressed as units of activity/mg protein. Inhibition (%) was expressed as percent decrease in GPDH activity against control (0%).

### 4.8. RNA Extraction and Real-Time RT-PCR

Total RNA was extracted using Total RNA Isolation Kit (NA017-0100, GeneDireX, Las Vegas, NV, USA) according to the manufacturer’s instructions. RNA quantity and purity were checked by spectrophotometric analysis at 260 and 280 nm. One microgram of total RNA was reverse transcribed into cDNA using GScript First-Strand Synthesis Kit (MB305-0050, GeneDireX). The lists of specific primers (Invitrogen, Carlsbad, CA, USA) were: GAPDH, Forward: 5′-GTATGACTCCACTCACGGCAAA-3′ (N3541G11), Reverse: 5′-GGTCTCGCTCCTGGAAGATG-3′ (N3541H01); PPAR-γ, Forward: 5′-TTTTCAAGGGTGCCAGTTTCGATCC-3′ (N3541H02), Reverse: 5′-AATCCTTGGCCCTCTGAGAT-3′ (N3541H03); C/EBPα, Forward: 5′-CGCAAGAGCCGAG ATAAAGC-3′ (N3541G05), Reverse: 5′-CACGGCTCAGCTGTTCCA-3′ (N3541G06); Sterol regulatory element binding protein 1 (SREBP/l c), Forward: 5′-GGAGCCATGGATTGCACAT-3′ (N3541H06), Reverse: 5′-GCTTCCAGAGAGGAGGCCAG-3′ (N3541H07).

The primers were added at final concentration of 250 nM to a 20 μL reaction mixture containing 4 μL of 5× OmicsGreen qPCR Master Mix and 1 μL of DNA template (stock conc. 1 μg/mL). The PCR (StepOnePlus™ Real-Time PCR System, Applied Biosystems™, Foster, CA, USA) conditions were denaturation at 90 °C for 15 s, annealing at 60–65 °C for 20 s, and elongation at 72 °C for 20 s in a cycle of 40 (OmicsGreen qPCR 5× Master Mix with ROX Dye, QE3931, Omics Bio, Taipei, Taiwan). The relative levels of gene expression were quantified using the ∆∆Ct method, which results in a ratio of target gene expression relative to equally expressed housekeeping genes (GAPDH) [36].

### 4.9. Western Blot Analysis

3T3-L1 adipocytes were harvested 8 days after the initiation of differentiation. Cells were incubated with 10–500 µM of test samples for 18 h at 37 °C in a humidified 5% CO_2_ incubator. Cells were collected and lysed in ice-cold lysis buffer (20 mM Tris-HCl (pH 7.4), 2 mM EDTA, 500 µM sodium orthovanadate, 1% Triton X-100, 0.1% SDS, 10 mM NaF, 10 µg/mL leupeptin and 1 mM PMSF). The protein concentration was estimated with the Pierce^TM^ BCA protein assay kit (Thermo Scientific) using bovine serum albumin as a standard. Proteins were fractionated on a SDS-PAGE gel and blotted for 2.5 h at 100 V. The proteins in the gel were transferred to a PVDF membrane. The membrane was blocked with 5% BSA in PBST (0.1% *v*/*v* Tween-20 in PBS, pH 7.2) for 1 h. β-actin (1:1000 dilution) and PPARγ (1:1000 dilution) of primary antibodies were used in this study. Goat anti-rabbit IgG-HRP and rabbit anti-rabbit IgG-HRP (Jackson, West Grove, Chester, PA, USA) were used as secondary antibodies, and ECL Prime (Advansta Inc., Menlo Park, CA, USA) reagent was used for developing. Membranes were incubated with primary antibody at 4 °C overnight and then with secondary antibody for 1 h. Membranes were washed in PBST for 10 min three times between each step. Blots were developed using the Western Bright ECL kit (Advansta Inc.), exposed to Gel Electrophoresis Documentation-Multi-function Gel Image system (Topbio Co., Taipei, Taiwan). The relative expression of proteins was quantified densitometrically using the Image J software (Wayne Rasband, Madison, WI, USA) and calculated according to the reference bands of β-actin.

### 4.10. HPLC/ESI-MS-MS Analysis

The HPLC/electrospray ionization (ESI) mass spectrometric analysis was performed on an Agilent 1260 Infinity HPLC system in connected with a 6420 mass spectrometer (Agilent Technologies, Santa Clara, CA, USA) in positive ionization mode as our previous reports [5,6] with minor modification. In brief, ethanolic extracts (10 mg/mL) of Djulis was filtered through 0.45 µm membrane filter before being injected into the analysis column of Symmetry C-18 column (2.1 × 150 mm, 3.5 μm particle size, Waters Corporation, Milford, Worcester, MA, USA), which was connected with a guard column (SecurityGuard C18 (ODS) 4 mm × 3.0 mm ID, Phenomenex Inc., Torrance, CA, USA) and was installed in a column oven set at 35 °C. The mobile phase consisted of two solvents: Solvent A (water containing 0.1% formic acid) and Solvent B (acetonitrile containing 0.1% formic acid). The flow rate during the elution process was set at 0.2 mL/min. A linear gradient elution was carried out with 20–30% B in 15 min, 30–95% B in 65 min and finally 95% B isocratic elution for 10 min. The absorption spectra of eluted compounds were scanned within 210 to 600 nm using the in-line PDA detector monitored at 280, 360 and 530 nm, respectively. The compounds having been eluted and separated were further identified with triple quadruple (QQQ) mass spectrometer in the operating parameters as follows: nitrogen used both as a drying gas at a flow rate of 9 L/min and as a nebulising gas at a pressure of 35 psi, drying gas temperature 300 °C, and a potential of 3500 V applied across the capillary, fragmentor voltage 90 V, and the collision voltage 15 V. Quadrupole 2 was applied for the scanning ions produced by nitrogen collision of ionized compounds in the range 100–800 *m*/*z* at a scan time of 200 ms per cycle. The identification of separated compounds was carried out by comparing their mass spectra provided with UV-Vis spectra and ESI-MS from our previous report [6] and those described in literatures and remarked in the Table 1.

### 4.11. Statistical Analysis

Data are expressed as mean ± SD, and ANOVA was conducted by using the SPSS software (SPSS Inc., Chicago, IL, USA). When a significant F ratio was obtained (*p* < 0.05) a post hoc analysis was conducted between groups by using a Duncan’s multiple range tests or a Dunnett’s test. *p*-Values of less than 0.05 were considered statistically significant.

## 5. Conclusions

In summary, the data presented in this study clearly shows EECF inhibited lipid accumulation. This mechanism may be attributed to down regulating the gene expression of PPARγ, C/EBPα and SREBP-1c, and thereby leading to the inhibition of adipose tissue formation and adipocyte function. The inhibitory effect of EECF on lipogenesis is related to bioactive compounds such as rutin, kaempferol and betanin presented in EECF. These results offer new insight into preventing adipogenesis and supplementation with EECF could be considered a potential therapeutic treatment to reduce the adipogenesis. In other words, EECF was a candidate for alleviating hyperlipidemia and/or against obesity. Meanwhile, this study provides positive indications that daily intake of Djulis may benefit to human health and lessen lipid accumulation. To verify this result, scientific trials in vivo are under-way.

## Figures and Tables

**Table 1:** Retention time, UV-Vis and Mass spectral characteristics of the ethanol extract of Djulis (*Chenopodiun formosaneum*).

Peak No.	Compound	*t*_R_ (min)	λ_max_ (nm)	[M + H]^+^	[M − H]^−^	Amount (mg/g) ^d^	References
1	protocatechuic acid derivatives	9.05	262, 234	285	283	7.328	
2	amaranthine ^b^	15.05	538	727	-	0.24	[9]
3	betanin ^a^	17.09	534, 274	551	-	0.74	
4	isobetanin ^b^	19.28	534, 236	551	-	0.28	[9]
5	quercetin derivatives	27.61	254, 354	889	-	4.29	
6	quercetin-3-*O*-rutinoside-7-*O*-rhamnoside	29.97	254, 354	757	755	7.49	[10]
7	quercetin-3-*O*-deoxy-hexose-*O-*hexose-*O*-pentoside	30.85	254, 352	743	-	1.86	[11]
8	camellianoside ^b^	31.26	268, 350	743	741	13.48	[12]
9	kaempferol derivative ^c^	32.34	268, 348, 236sh	697	695	1.42	
10	kaempferol-3-*O*-[6′″-*p*-coumaroyl-glucosyl-β-(1→4)-rhamnoside] ^b^	32.77	268, 349, 246sh	741	739	3.09	[13]
11	quercetin-3-*O*-2′′-(6′′-*p*-coumaroyl)-glucosylrhamnoside	33.09	254, 354	757		5.54	[14]
12	rutin ^a^	33.61	349, 267, 226	611	609	25.89	
13	unknown	33.90	220, 268, 352	291	-	6.61	
14	20-hydroxyecdysone	34.99	246, 224sh	481	-	8.06	[15]
15	unknown	35.50	222, 328	479	-	1.81	
16	kaempferol-3,7-di-*O*-rhamnoside ^c^	36.07	350, 266, 234	579	577	1.99	[16]
17	kaempferol-3-*O*-rutinoside ^a^	37.06	267, 350, 235sh	595	593	0.90	
18	unknown	37.89	222, 252, 340	625	-	3.25	
19	unknown	42.25	222, 314	453	-	1.70	
20	unknown	43.70	222, 312	677	675	4.04	

^a^ The identification was further confirmed by comparison with the authentic compound. ^b^ Compounds were tentatively identified according to mass spectra and the matched data from the literature as shown in following 1–8. ^c^ Compounds were limitedly identified from mass spectra and UV-visible absorbance spectra. ^d^ Peaks 2, 3 and 4 were quantified as equivalent to betanin and all the others were quantified as quercetin (three replicates for all compounds) based on the amount of mg/g aqueous extract of Djulis.
